# Product Development of High-Dose Ambroxol HCl Capsules for an n-of-1 Clinical Trial Involving Dutch Patients with Gaucher Disease Type 3

**DOI:** 10.3390/pharmaceutics17040417

**Published:** 2025-03-25

**Authors:** Hoang Lan Le, Natalja Bouwhuis, Carla E. M. Hollak, Abraham J. Wilhelm, Anne-Loes E. Gerards, Yuma A. Bijleveld, Eleonora L. Swart

**Affiliations:** 1 Department of Pharmacy and Clinical Pharmacology, Amsterdam UMC Location University of Amsterdam, 1105 AZ Amsterdam, The Netherlands; 2 Medicine for Society, Platform at Amsterdam UMC Location University of Amsterdam, 1105 AZ Amsterdam, The Netherlands; 3 Gastroenterology Endocrinology Metabolism Research Institute, Amsterdam UMC Location University of Amsterdam, 1105 AZ Amsterdam, The Netherlands; 4 Department of Endocrinology and Metabolism, Amsterdam UMC Location University of Amsterdam, 1105 AZ Amsterdam, The Netherlands

**Keywords:** ambroxol hydrochloride, product validation, good manufacturing practices (GMPs), product development, active pharmaceutical ingredient (API), Gaucher disease, rare diseases, n-of-1 clinical trial, drug product specification, pharmaceutical quality

## Abstract

**Background/Objectives**: Ambroxol hydrochloride (AMB) is a promising chaperone for treating neurological manifestations in Gaucher disease type 3 (GD3). The Amsterdam University Medical Center planned to conduct an n-of-1 clinical trial using high-dose AMB (25 mg/kg/day). As an adequate commercial AMB formulation is unavailable for this high target dosage, we aimed to develop high-dose AMB capsules and assess the formulated capsule’s quality. **Methods**: AMB API was sourced and tested according to the requirements of the European Pharmacopoeia. Capsule formulations of 75 mg and 200 mg AMB were developed. Drug product specifications were set following international guidelines (ICH Q6A) and the European Pharmacopoeia. Analytical methods were developed and validated, and three validation batches of each capsule strength were produced and analyzed. **Results**: The contents and the Acceptance Values (AVs) of the initial AMB batches (both strengths) varied between 89.1% to 92.7% (specification: 90% to 110%) and 12.4 to 17.6 (specification ≤ 15.0), respectively, indicating non-uniform AMB distribution. Consequently, the production of 200 mg capsules was discontinued, and modifications were made to the 75 mg capsule formulation, followed by the production of three optimized 75 mg validation batches. These batches met the specified criteria, with an AMB content and AV values ranging from 93.9% to 96.5% and 12.4 to 14.9, respectively. Furthermore, rapid dissolution profiles were observed (>80% dissolution within 15 min). No degradation products or microbiological impurities were detected after production. **Conclusions**: The optimized formulation of 75 mg AMB capsules formulated within the hospital pharmacy setting resulted in qualitative and uniform capsules which can be used in clinical trials.

## 1. Introduction

Gaucher disease type 3 (GD3) is a rare, autosomal recessive lysosomal storage disease. Gaucher disease is characterized by a deficiency in the enzyme glucocerebrosidase (GCase) caused by mutations in the *GBA1* gene. Due to GCase deficiency, the substrate glucocerebroside (GL-1) accumulates primarily in macrophages, mainly resulting in systemic manifestations, such as anemia, hepatosplenomegaly, bone disease, and growth retardation [1,2,3]. These non-neurological manifestations characterize type 1 Gaucher disease (GD1), which can be treated with enzyme replacement therapy (ERT) or substrate reduction therapy (SRT) [4,5]. Neuronopathic manifestations characterize type 2, which is early lethal, and type 3.

In GD3, less progressive but variable neurological manifestations, in addition to the systemic manifestations, are present. The addition of neurological complications is believed to be the result of the formation of glucosylsphingosine (Lyso-GL1), the deacylated form of GL-1. Accumulation of Lyso-GL1 in the brain results in neurological symptoms, such as seizures, ataxia, and cognitive decline [6]. Treatment of GD3 is particularly challenging, as there is no therapeutic option available that can cross the blood–brain barrier (BBB) to treat these neurological manifestations. This significantly impairs the patient’s quality of life.

Maegawa, et al. have identified ambroxol hydrochloride (AMB), a mucolytic agent for the treatment of respiratory diseases, as a potential drug for the treatment of GD3 as it increases GCase activity [7]. AMB is capable of crossing the BBB, making it a promising candidate for the treatment of the neurological symptoms in patients with GD3. The efficacy of high-dose AMB (25 mg/kg/day, max 1300 mg/day) in GD3 has been reported in several observational studies without significant adverse effects [8,9,10,11,12,13,14,15]. Despite promising reports, data are still lacking on the effectiveness and safety of high-dose AMB [16]. The rarity of GD3 complicates collecting robust data in randomized controlled trials. Additionally, AMB is not available in many countries, limiting both patient access and research opportunities. In the Netherlands, only the 20 mg AMB lozenge Mucoangin^®^ is available [17]. These low-dose lozenges are unsuitable for high-dose AMB clinical trials as participants would need to ingest up to 65 lozenges per day. Furthermore, the development of placebo lozenges is another complicating and high-priced factor for the use in placebo-controlled clinical trials.

The aim of this study was to develop high-dose AMB capsules in a hospital pharmacy setting that are suitable for use in investigator-initiated clinical trials and assess their pharmaceutical quality. This paper describes the initial product validation of 75 mg and 200 mg AMB capsules, and a second product validation of the optimized formulation of 75 mg AMB capsules.

## 2. Materials and Methods

### 2.1. Raw Materials and Chemicals

AMB active pharmaceutical ingredient (API) was sourced and purchased through supplier Duchefa Farma B.V. (Haarlem, The Netherlands) from manufacturer Ven Petrochem & Pharma Pvt Ltd. (Mumbai, India). The excipients lactose monohydrate and silica (colloidal anhydrous) were purchased from Fagron NL B.V. (Capelle aan den IJssel, The Netherlands). Clear hard gelatin capsules (sizes 0 and 2) were purchased from Spruyt Hillen (Capelle aan den IJssel, The Netherlands). For packaging, pharmaceutical grade white 100 mL Duma^®^ HDPE Twist-Off bottles with corresponding PP screw caps were procured from Aluglas Packaging Group Europe (Uithoorn, The Netherlands). The capsule filling Capsicard^®^ machine was purchased from Spruyt Hillen (Capelle aan den IJssel, The Netherlands).

The manufacturer of AMB API holds a Certificate of Suitability (CEP), confirming that the quality of the API is suitably controlled in accordance with the current version of European Pharmacopoeia (Ph. Eur.) individual Monograph ‘Ambroxol Hydrochloride’ (Ph. Eur. 1489) [18]. The excipients all complied to the specifications of the Ph. Eur. [19,20].

The reference standards for the analysis of AMB API and capsules were Ph. Eur. grade and were purchased from Sigma Aldrich (Zwijndrecht, The Netherlands).

### 2.2. Production of Validation Batches AMB Capsules

Capsules containing 75 mg and 200 mg AMB were produced by the Pharmacy Department at Amsterdam UMC following Good Manufacturing Practices (GMPs) guidelines and National production guidelines (LNA Capsule procedure) [21,22]. The capsule strengths were based on the predicted AMB use of Gaucher patients treated at the Amsterdam UMC hospital.

AMB has strong cohesive- and poor flow properties. Silica was added as a glidant to improve the flow properties and lactose monohydrate was used as a diluent. For the initial formulations of 75 and 200 mg AMC capsules, silica was added in an amount equal to 1% of the amount of AMB. A graduated cylinder was used to fill lactose monohydrate to the desired volume depending on the size of the capsules. The dry powder mixture was manually homogenized using a mortar and pestle. The weighing and homogenizing of the raw materials were performed per portion of 100 capsules before filling 100 capsules at a time using a manual capsule filling machine. Three validation batches were produced by different qualified pharmacy technicians on different days for both doses.

In-process controls were performed on 10 random selected capsules of every portion of 100 capsules produced to evaluate the mean weight deviation and intra-batch weight variability. The mean weight deviation was evaluated by calculating the deviation of the mean capsule weight from the theoretical weight. Intra-batch weight variability was evaluated by calculating the relative standard deviation (RSD). The limits were set according to the national guidelines for the production of capsules, derived from the Ph. Eur. (mean weight deviation < ±3.0%, and RSD < 4.0% for capsule contents < 300 mg and RSD < ±3.0% for capsule content ≥ 300 mg) [22]. Upon meeting the specified criteria, all batch portions were mixed together prior to being packaged per 50 capsules into bottles to ensure a homogeneous batch. Portions that did not comply with the in-process controls were destroyed and replaced with a new portion of 100 capsules that did comply.

The product validation for 75 mg capsules was performed twice. The second product validation was performed after the initial product validation was deemed invalid and after the production process and capsule formulation was optimized. The capsule formulation of 75 mg capsules was adjusted to include 200 mg of silica as a fixed amount, which is equivalent to approximately 1% of the total powder mixture (unlike the initial formulation where 1% of the amount of AMB was added). Also, to reduce the number of crucial steps in the production process, minimizing the risk of powder loss, and optimizing the homogeneity, weighing, and homogenizing of the raw materials for the entire batch (1000 capsules) was performed instead of per portion of 100 capsules. Subsequently, the powder mixture was divided into 10 portions by weight (each for production of 100 capsules) and capsules were filled with the manual capsule filling system.

The product validation for 200 mg capsules was discontinued as adding an increased amount of silica did not improve capsule quality as observed during test preparations. It was decided that patients would be able to receive adequate therapy with only 75 mg AMB capsules.

### 2.3. Product Specifications

Product specifications were set according to applicable Ph. Eur. monographs and ICH guidelines [18,23,24,25,26]. Quality Control analyses were performed on every validation batch and included the following: identity, related substances, assay content, uniformity of dosage units (UDUs), dissolution, and microbiology. See Table 1 for all specifications and acceptance limits. According to the Ph. Eur., the UDU can be expressed using two methods depending on the amount of API and the proportion of API to the total powder mixture [27]. CU was chosen as the standard method although MV was allowed in some batches.

### 2.4. Analytical Methods

No pharmacopeial monograph is available for the finished AMB capsules; hence, a non-compendial assay method partly adapted from Ph. Eur. was developed [18]. High pressure liquid chromatography with UV spectrophotometry (HPLC-UV) was used to identify and quantify AMB and its related substances in the capsules using a Thermo Scientific Dionex UltiMate 3000 RS (Thermo Fisher Scientific, Breda, The Netherlands) with a Luna C18 column (250 mm × 4 mm, 5 µm, Phenomenex) and a UV-2600 UV-VIS spectrophotometer (Shimadzu, Kyoto, Japan). The HPLC-UV method was fully validated with a calibration curve linearity (R^2^) of 0.998 between 70–130 µg/mL (criteria: ≥0.990). Method accuracy and imprecision at 70%, 100%, and 130% of the nominal concentrations were 100.3% and 0.72%, 99.6% and 0.46%, and 100.0% and 0.39%, respectively (criteria accuracy, 97.5–102.5%; and imprecision, <2.5%).

From each batch, 10 capsules were collected as a sample for quality control. The capsules were dissolved in 20 mL of ultra-pure water using an ultrasonic bath. The solution was centrifuged and subsequently diluted 3.75 times (75 mg capsules) or 10 times (200 mg capsules) with ultra-pure water, followed by an additional 10-fold dilution with mobile phase. The mobile phase comprised of 50% acetonitrile and 50% di-ammonium hydrogen phosphate buffer (pH 7). The HPLC flow-rate was 1 mL/min and the temperature of the column oven was set to 25 °C. The samples were analyzed at a detection wavelength of 248 nm. The AMB content was calculated using a calibration curve constructed from 70%, 100%, and 130% AMB reference standards. For UDU, if needed, 20 additional capsule samples were collected and quantified for a total of 30 samples.

For dissolution testing, multiple point sampling was performed on the initial formulations at 15, 30, 45, and 60 min to generate a dissolution profile for each validation batch. Single point sampling at 30 min was used for the second product validation based on the dissolution profile of the initial formulations. From each batch, 7 capsules were collected as a sample for the dissolution test. The dissolution testing was performed using the basket apparatus Sotax AT7 Smart (PMT Benelux, Kampenhout, Belgium) with a rotation speed of 75 rpm. The dissolution medium consisted of 900 mL 0.1 M HCl. A sample of 15 mL was taken from the dissolution medium. The dissolved AMB content was measured with UV-spectrophotometry at 310 nm instead of 248 nm, because of differences in solvents compared to the HPLC-UV method. The UV-spectrophotometry method was fully validated with R^2^ of 1.000 between 0–0.21 mg/mL (criteria: ≥0.996), accuracy of 100.1% (criteria: 99.0–101.0%), and imprecision of 0.18% (criteria: <1.0%).

### 2.5. Stability Study

The remaining bottles of the validation batches were placed in a climate chamber under specific conditions for stability testing for 9 months according to the ICH guideline Q1A (R2) [28]. The conditions were 25 °C ± 2 °C/60% relative humidity (RH) ± 5% RH for the long-term stability and 40 °C ± 2 °C/75% RH ± 5% RH for the accelerated stability study. However, this study was discontinued as result of the change in capsule formulation. The results will, therefore, not be further discussed in this paper. Reference is made to this study, as the results at t = 3 months (t = 3) compared to the results of the initial product validations (t = 0) will be described in this paper.

### 2.6. Out-of-Expectiation Investigation

A product validation was considered invalid when at least one of the three validation batches contained any results out of specification (OOS), but also unexpected results (out-of-expectation, OOE). The initial product validation resulted in OOS for the AMB content (<90%) and UDU (>15) for both capsule strengths (75 mg and 200 mg). Also, during the following investigation, an increase in AMB content after 3 months of storage in the stability chamber during both the long-term and the accelerated stability studies was identified. To clarify the deviations, additional analyses were conducted, focusing on the properties of AMB API, the production process, and the analysis method. The relevant analyses are detailed below.

#### 2.6.1. Properties of AMB API

The possible hygroscopicity of AMB API was investigated, as it is a salt. Hygroscopicity might influence the weight of AMB API during storage. This was evaluated with the loss on drying (LOD) method at 105 °C for 2.5 h [18]. The samples were taken from a 1-year-old container, which was used for the production of all validation batches, and a newly purchased unopened container. The LOD of both samples were compared.

#### 2.6.2. Error and Variations in Analytical Method

All analysis of the initial product validations were carried out by the same laboratory technician, which could have influenced the results. Particularly, the AMB content results were consistently low, which is in contrast to the content values reported with the dissolution testing (see Section 3). To rule out potential human error, additional analyses were performed. First, two other laboratory technicians each analyzed 10 randomly selected capsules from the same bottle of 75 mg capsules (initial formulation), which was stored for 3 months (t = 3) for the long-term stability study. The mean AMB contents obtained by both laboratory technicians were compared with each other and with the mean AMB content from the initial analysis immediately after the production (t = 0) of this validation batch. Secondly, one laboratory technician analyzed 10 randomly selected capsules from the same bottles of 200 mg capsules (initial formulation) used at t = 0 and at t = 3. These bottles were stored in the laboratory at room temperature after the initial analysis. The results were compared to the mean AMB content from the initial analysis at t = 0 and t = 3 of this validation batch.

## 3. Results

### 3.1. In-Process Controls of the Produced AMB Formulations

#### 3.1.1. The Initial Formulations

Three batches of 75 mg capsules were produced, each consisting of 800 to 1400 capsules. Ten to fourteen portions were produced per batch. In total, three portions of 100 capsules did not meet the specifications of the in-process controls. The failed portions of 75 mg included RSD’s of 4.13%, 4.22%, and 5.18% (specification: <4.0%). These portions were destroyed and two were replaced by new portions that did comply.

The three batches of 200 mg capsules were produced, each consisting of 800 to 1100 capsules. In total, five portions did not meet the specifications of the in-process controls. The failed portions of 200 mg included RSD’s between 3.08% to 3.21% (specification: <3.0%). These portions were destroyed and replaced by new portions that did comply. The results of the in-process controls of the initial batches are shown in Table 2.

#### 3.1.2. The Optimized Formulation

Based on the high number of portions failing the in-process controls (see Table 2), validation batches not meeting the specification for content and UDU (see Table 1), and the OOE investigation, of which the results are described later in this paper, the formulation of the 75 mg capsules was optimized as described in Section 2.2. Consequently, three new validation batches of 75 mg capsules were produced, each batch consisting of 1000 capsules. No portion failed the in-process controls after optimization (see Table 2).

### 3.2. Product Validation

#### 3.2.1. Product Validation of the Initial Formulations

**Figure 1:**
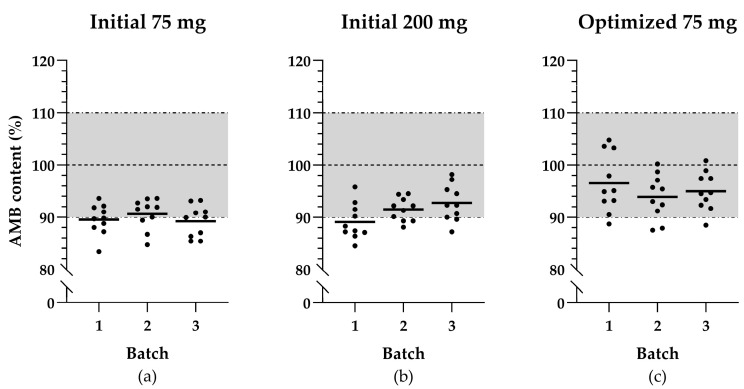
Scatter plots illustrating ambroxol hydrochloride (AMB) content distribution of the selected capsule samples across validation batches: (**a**) initial capsules of 75 mg AMB; (**b**) initial capsules of 200 mg AMB; (**c**) optimized capsules of 75 mg AMB. The black lines indicate the mean of the AMB content per batch, the horizontal dashed line represents the target content for the produced capsules, and the dashed-and-dotted lines together with the grey area represent the acceptable range for mean capsule content.

All results are listed in Table 1. Only three of the six initial validation batches complied with all specifications. With the HPLC-UV assay, an average AMB content in both 75 mg and 200 mg capsules of 90.4 ± 1.4% was measured, which is generally low (see Figure 1a,b). Moreover, the assay content of two batches of the initial 75 mg capsules and one batch of the initial 200 mg capsules fell below 90% and were, therefore, OOS (see Table 1). These three validation batches also exceeded the specification of AV ≤ 15.0.

**Figure 2:**
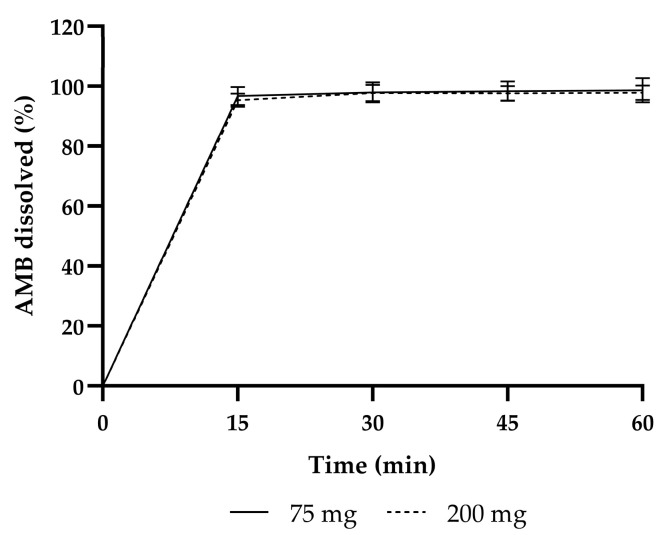
Dissolution profiles of the initial product validation of 75 mg and 200 mg AMB.

The capsules had rapid dissolution profiles, with complete dissolution within 15 min, meeting the specification of >80% at 30 min (see Figure 2). The average release per batch at 30 min are listed in Table 1.

Interestingly, during dissolution testing, the average AMB content determined by UV-VIS was higher (75 mg and 200 mg together: 97.8 ± 1.8%) than the average AMB content measured by the HPLC-UV (75 mg and 200 mg together: 90.4 ± 1.4%).

#### 3.2.2. Product Validation of the Optimized 75 mg Formulation

The three validation batches of the optimized 75 mg capsule formulation met all predefined specifications, including the average capsule contents and AV’s. The distribution of all individual capsule contents is shown in Figure 1c. The dissolution profile testing was replaced by single-point measurements at 30 min. The average dissolution values of the optimized validation batches corresponded more closely with the AMB content determined by the HPLC-UV assay (see Table 1).

### 3.3. Out-of-Expectation Investigation

#### 3.3.1. Hygroscopicity of AMB

The water content was 0.11% in the one-year-old container and 0.07% in the newly purchased unopened container. Both were well below the limit of 0.5% [18].

#### 3.3.2. Error and Variations in Analysis Method

**Figure 3:**
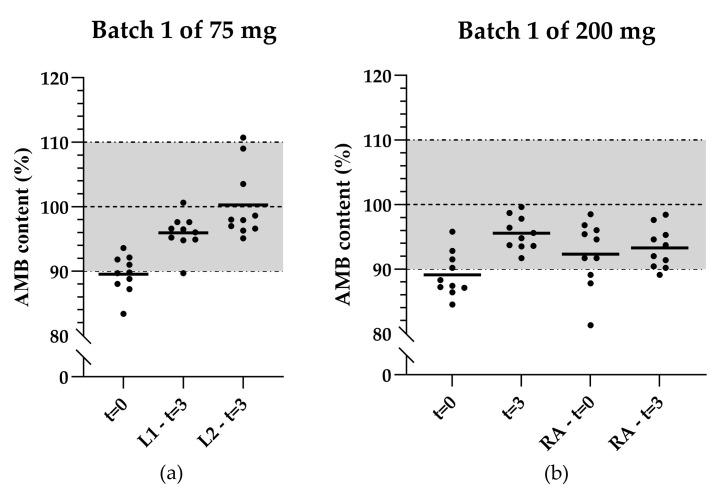
Scatter plots illustrating the content distribution of the initial product validation and after 3 months of storage of (**a**) 75 mg AMB capsules in the same bottle analyzed by two laboratory technicians; (**b**) 200 mg AMB capsules reanalyzed by another laboratory technician. The black lines indicate the mean of 10 capsules, the horizontal dashed line represents the target concentration, and the dashed-and-dotted lines together with the grey area represent the acceptable range for mean capsule content. t = 0: Analysis during the initial product validation. t = 3: Analysis after 3 months of storage. L1: Laboratory technician 1. L2: Laboratory technician 2.

The AMB content of the 75 mg capsules (initial formulation) at t = 3 analyzed by two laboratory technicians from one bottle was higher compared to t = 0 (see Figure 3a). At t = 3, average AMB contents of 96.0% and 100.3% were found, whereas an average content of 89.5% was found at t = 0.

The increase in average AMB content at t = 3 compared to t = 0 was also observed in the 200 mg capsules (initial formulation). The average AMB content at t = 0 and t = 3 originally were 89.1% and 95.5%, respectively. Reanalysis of the same bottles by another laboratory technician resulted in average AMB contents of 92.3% and 93.3%, respectively (see Figure 3b).

## 4. Discussion

This study aimed to investigate the pharmaceutical quality of the AMB capsules developed by our pharmacy through the validation of the finished product. Two full product validations were conducted prior to commencing clinical trials, to ensure that the formulation met quality standards. The initial product validations of the 75 mg and 200 mg AMB capsules did not comply with all predefined specifications and were considered invalid. The second product validation of the optimized 75 mg capsules complied with all predefined specifications, indicating that the capsules were of high quality after the formulation was improved. The improvements consisted of increasing the amount of silica to increase the flow properties of the powder mixture and minimizing the amount of weighing and homogenizing steps to optimize the homogeneity. These improvements resulted from the OOE investigation following the initial product validation.

The initial product validations of 75 mg and 200 mg failed to meet two of the six predefined specifications: capsule content and UDU. The capsule content must not deviate by more than 10% of the content stated on the label according to the Dutch Medicines Act (Geneesmiddelenwet) and the UDU must result in AV ≤ 15.0 according to the Ph. Eur. [27,29]. Three of the six validation batches resulted in assay contents under the lower limit of 90%. The same three batches resulted in AV exceeding 15.0. Additionally, the capsule contents obtained from the HPLC-UV assay (90.4 ± 1.4%) were consistently significantly lower compared to the amount of AMB released in the dissolution tests (97.8 ± 1.8%). These discrepancies raised concerns regarding the reliability of the capsule content results as the analytical method had been thoroughly validated. In the OOE investigation, several potential causes for the deviations were explored, focusing on the properties of AMB API, the production process, and the analytical method.

The most plausible cause for the deviations was an uneven content distribution of AMB in the capsules. Multiple findings indicated this possibility, although not always unambiguously. The first indication was the high number of portions that failed in-process controls because of high RSD. High RSDs, despite the average weight being within specification, generally indicate insufficient flowability or an insufficient final volume of the powder mixture, resulting in the uneven distribution of the powder mixture over the capsule [30]. The insufficient flowability of the powder mixture was most likely the cause, as the flow properties improved after increasing the amount of silica during test preparations. The capsule formulation was, therefore, revised for the second product validation. To identify suboptimal capsule formulations at an earlier stage of product validation, we recommend adding a maximum number of failed portions as an in-process control to the product validation design. The cause for these failures should always be evaluated before laboratory analysis of the end product.

The second indication was the overly high AV values and low AMB content during the analysis of three validation batches. High AV values indicate high variation in capsule content and thus a non-homogeneous powder mixture [30]. To address this problem, we optimized the formulation by minimizing the amount of weighing and homogenizing steps. The initial production process required repeating weighing and mixing raw materials for each portion of 100 capsules, making it time-consuming and leading to the deterioration of the homogeneity over time. Weighing and homogenizing the raw materials for the entire batch may contribute to production of a more homogeneous mixture.

The third indication was an unexpected increase in AMB content for both 75 mg and 200 mg capsules at t = 3 of the stability studies compared to the initial result at t = 0. Uneven AMB distribution over the capsules within a bottle may have caused more capsules containing low AMB content in the analysis at t = 0, possibly due to chance.

Though the indications did confirm the theory of uneven content distribution of AMB across the capsules, the results did not align with the consistent difference in AMB content observed in the content assay compared with the dissolution tests. This discrepancy can be explained by noting that the dissolution analysis method was not developed for the precise quantification of AMB content. This method requires the analysis of a 15 mL sample taken from a 900 mL solution. The content is back-calculated, introducing a significant margin of error. This test determines the dissolution rate, but the values cannot be compared with the assay results. In addition, the OOE investigation demonstrated that other variables besides the uneven content distribution were not the underlying cause for the deviations observed. No significant errors were found in the production- or analysis process, since all involved personnel were qualified laboratory or pharmacy technicians, and the production and analyses were found to be well executed. Furthermore, AMB was not hygroscopic and, therefore, no influence of adsorbed water was expected in the weighing of AMB during the production.

Improving the formulation and production process resulted in high-quality capsules, which ensure patient safety and maintaining the integrity of the study’s results. The improved formulation of 75 mg is a practical solution for investigator-initiated clinical trials with AMB. Additionally, it is also beneficial for regular healthcare practices when effectiveness of high-dose AMB is observed in children with GD3 and no commercially available product is available or accessible.

This study is significant in the field of rare diseases, as it highlights that the process of drug development for investigator-initiated clinical trials is not always straightforward. One must account for the possibility of unforeseen complexities during the starting phase of a study design, particularly when conducting research in small patient populations for which there is little budget, such as investigator-initiated trials in rare diseases. By sharing our experience, we aim to provide pharmacists with a starting point for their AMB product development and, in doing so, encourage further research not only on repurposing AMB in GD3, but also on AMB in other diseases, such as Parkinson’s disease [31].

## 5. Conclusions

In conclusion, high-dose (75 mg) AMB capsules of high quality were developed by our hospital pharmacy. Our study proves that these capsules can be effectively formulated in a hospital pharmacy setting for use in clinical trials in GD3 patients. However, caution needs to be taken due to the poor flow properties of AMB raw material. Improving the flow properties with sufficient excipients is essential to obtain high-quality preparations. With this paper, we provide guidance to pharmacies for establishing their own production process for AMB capsules to treat their patients in need. This form of collaboration could improve the availability of AMB for research and for patients suffering from the rare type 3 Gaucher disease.

## Figures and Tables

**Table 1:** Product specifications and results of the initial product validation of 75 mg and 200 mg AMB capsules and the optimized product validation of 75 mg AMB capsules.

		Initial Product Validation 75 mg			Initial Product Validation 200 mg			Optimized Product Validation 75 mg		
Tests	Acceptance Limit	Batch 1	Batch 2	Batch 3	Batch 1	Batch 2	Batch 3	Batch 1	Batch 2	Batch 3
Identity	Complies	Complies	Complies	Complies	Complies	Complies	Complies	Complies	Complies	Complies
Related substances (%) Unspecified impurities Total impurities	<0.1 <0.3	<0.1 <0.3	<0.1 <0.3	<0.1 <0.3	<0.1 <0.3	<0.1 <0.3	<0.1 <0.3	<0.1 <0.3	<0.1 <0.3	<0.1 <0.3
Mean content (% of labelled content)	90–110%	89.5	90.6	89.2	89.1	91.5	92.7	96.5	93.9	95.0
Uniformity of dosage units (Content Uniformity)	AV ≤ 15.0	16.0	15.0	16.5	17.6	12.4	14.2	14.5	14.9	12.4
Dissolution	≥80% at 30 min	100.5%	97.2%	96.0%	96.5%	99.4%	97.3%	95.0%	99.5%	96.3%
Microbiology TAMC TYMC *E. coli*	<10^3^ CFU/g <10^2^ CFU/g Absent	<10 <10 Absent	<10 <10 Absent	<10 <10 Absent	<10 <10 Absent	<10 <10 Absent	<10 <10 Absent	<10 <10 Absent	<10 <10 Absent	<10 <10 Absent

AV, acceptance value; TAMC, total aerobic microbial count; TYMC, total yeast and mold count. Shaded grey values represent non-compliance with the prespecified specifications.

**Table 2:** Results of the in-process controls of the capsule formulation.

Dose	Batch	Batch Size Meeting IPC (Caps)	Number of Portions Failed IPC	Capsule Content * (mg)	Mean Weight Deviation * (%) (<±3.0%)	RSD * (%) (75 mg: <4.0% 200 mg: <3.0%)
75 mg (initial)	1	1400	0	222.6 ± 14.3	−0.52 ± 0.70	2.23 ± 0.49
	2	900	1 ^†^	206.7 ± 10.0	−1.25 ± 0.59	2.38 ± 0.80
	3	800	2 ^†^	199.0 ± 5.0	−0.14 ± 1.38	2.58 ± 0.33
200 mg (initial)	1	1100	3 ^†^	303.8 ± 9.6	−1.19 ± 0.45	2.13 ± 0.30
	2	800	1 ^†^	298.4 ± 3.6	−1.37 ± 0.86	2.52 ± 0.41
	3	800	1 ^†^	328.0 ± 23.9	−0.06 ± 1.21	1.56 ± 0.28
75 mg (optimized)	1	1000	0	259.1 ± 2.6	−0.94 ± 1.00	2.37 ± 0.54
	2	1000	0	262.9 ± 1.1	−1.73 ± 0.53	1.64 ± 0.45
	3	1000	0	284.4 ± 1.8	−1.94 ± 0.62	1.86 ± 0.41

75 mg and 200 mg (initial): results of the initial formulation of 75 mg and 200 mg capsules. 75 mg (optimized): results after modifying the formulation and the production method of 75 mg capsules. Caps: capsules. IPC: in-process controls. RSD: relative standard deviation. * Values reported represent the mean and standard deviation (SD) of all in-process control results for each batch. ^†^ Results of the in-process controls of these portions are not included in this table.

## Data Availability

The original contributions presented in this study are included in the article. Further inquiries can be directed to the corresponding author.
